# Association Study of Reported Significant Loci at 5q35.3, 7p14.3, 13q14.1 and 16p12.3 with Urolithiasis in Chinese Han Ethnicity

**DOI:** 10.1038/srep45766

**Published:** 2017-03-31

**Authors:** Lujia Wang, Chenchen Feng, Guanxiong Ding, Xiaoling Lin, Peng Gao, Haowen Jiang, Jianfeng Xu, Qiang Ding, Zhong Wu

**Affiliations:** 1 Department of Urology, Huashan Hospital, Fudan University, Shanghai 200040, PR China; 2 Institute of Urology, Fudan University, Shanghai 200040, PR China; 3 Center for Genomic Cancer Research, North Shore University Health System, Evanston, Illinois, USA

## Abstract

In this study, we aimed to validate the association of 8 reported significant loci at 5q35.3, 7p14.3, 13q14.1 and 16p12.3 with urolithiasis in Chinese Han population. We performed case-control association analysis using 624 patients with nephrolithiasis and 1008 control subjects. We selected single-nucleotide polymorphism (SNPs) including rs12654812 and rs11746443 from 5q32.3; rs12669187 and rs1000597 from 7q14.3; rs7981733, rs4142110 and rs17646069 from 13q14.1 and rs4293393 from 16p12.3 which were previously reported to be associated with nephrolithiasis. We found none of these eight reported SNPs were significant associated with urolithiasis risk in Chinese Han population, which suggested that differences could exist in the mechanisms of calcium urolithiasis between Chinese and Japanese Ethnics. The A allele of rs12669187 was significantly correlated with increased level of serum magnesium. The C allele of rs1000597 was associated with higher levels of serum creatinine, uric acid, calcium and lower urine pH level. The T allele of rs4142110 was correlated with higher levels of serum magnesium, phosphorus, and lower AKP level. The G alleles of rs4293393 was associated with higher serum CO_2_ level. The risk alleles of these SNPs were proved to be associated with the electrolytes metabolism that may result in the formation of urolithiasis.

Urolithiasis is one of the most frequent disorders affecting almost all populations. It is a worldwide problem that is associated with substantial health and socioeconomic burdens. Epidemiological studies have reported that the prevalence of urolithiasis is about 7–15% in male and 3–6% in female^1^, and calcium nephrolithiasis represents 70–85% of cases^2^. The cause of urolithiasis is considered to be multifactorial, including but not limited to diet, ethnic, climate, and genetic factors. In recent years, the influence of genetic factors on urolithiasis has been receiving increased attention^3^,^4^. Studies in families and twin pairs have revealed that urolithiasis is inherited with a non-medelian transmission pattern involving multiple genes, indicating a pivotal role of genetic factors in its etiology^5^. Nowadays, the study of single nucleotide polymorphisms (SNPs) has been used as a tool for mapping genes that associated with variety of urolithiasis.

In 2012, a genome-wide association study (GWAS) on nephrolithiasis has been performed in the Japanese population, including 5892 nephrolithiasis cases and 17809 controls. They have identified three novel susceptible loci for nephrolithiasis at 5q35.3 (*RGS14*-*SLC34A1*-*PFN3*-*F12*), 7p14.3 (*INMT*-*FAM188B*-*AQP1*) and 13q14.1 (*DGKH*)^6^. In order to evaluate the role of these genetic factors, a validation study has been performed in 2013 by using an independent Japanese cohort, including 601 nephrolithiasis and 201 control subjects. Their findings elucidate the significance of genetic variation at these 3 loci in nephrolithiasis in Japanese population^7^. In 2014, an association study of *DGKH* genetic polymorphisms with calcium oxalate nephrolithiasis has been performed in Chinese population, and their findings also implicate a link between nucleotide variant of *DGKH* and nephrolithiasis^8^. Because of the similarity in ethnicity between the Japanese and Chinese Han populations, we try to validate the significance of genetic variation at these three loci in Chinese Han population. Moreover, since a SNP next to *UMOD* gene (16p12.3) has been proved to be associated with nephrolithiasis in a GWAS in Icelandic and Dutch stone-formers^9^, we have included it in our study as well. Hence, we have selected those eight single-nucleotide polymorphism (SNPs) including rs12654812 and rs11746443 from 5q32.3; rs12669187 and rs1000597 from 7p14.3; rs7981733, rs4142110 and rs17646069 from 13q14.1 and rs4293393 from 16p12.3 in our study.

## Material and Methods

### Ethics statement

The study protocol conformed to the Declaration of Helsinki. All written informed consents were obtained from all participants before conducting this study. This research protocol was approved by Huashan Institutional Review Board of Fudan University, and the experiments on human subjects were performed in accordance with relevant guidelines and regulations.

### Subjects

In total, clinical and demographic data of 624 unrelated Chinese patients treated for urolithiasis, including kidney stones and ureteral stones (441 males and 183 females, mean aged 24.42 ± 3.35 years) at Huashan Hospital of Fudan University between April 2011 and February 2015 are shown in detail in Table 1. Patients with urolithiasis secondary to known causes, such as chronic kidney disease (CKD), chronic diarrhea, renal tubular acidosis, gout, primary and secondary hyperparathyroidism, osteoporosis, or cancer were excluded. Patients with radioparent stones, including struvite, cystine and uric acid stones were also excluded. We also excluded patients with history of medications that affected urinary calcium excretion or taking vitamin D and/or calcium supplements. The control group consisted of 1008 subjects were age/gender matched individuals without a history of urolithiasis or a family history of urinary stone disease. All the patients with urolithiasis and the control subjects were of the same racial, ethnic, geographical and environmental strata. Urolithiasis was diagnosed clinically either with plane radiography of kidney-ureter-bladder (KUB) or non-enhanced computed tomography (CT) scan.

We assessed the effect of genetic variations on serum calcium, sodium, potassium, magnesium, phosphorus, creatinine, urea, uric acid, alkaline phosphatase (AKP), γ-glutamyl transferase (γGT), albumin, total protein, carbon dioxide (CO_2_), urine specific gravity, pH, glomerular filtration rate (GFR), and body mass index (BMI). Laboratory measurements were performed within 2 weeks preoperatively. GFR was calculated by 99mTc-DTPA dynamic SPECT/CT volumetry.

### SNP selection and genotyping

We selected a total of eight single-nucleotide polymorphisms (SNPs) at four loci on 5q35.3, 7p14.3, 13q14.1, and 16p12.3, which were previously reported to be significantly associated with nephrolithiasis^6^,^7^,^8^,^9^. We genotyped these SNPs on the MassARRAY iPLEX platform (Sequenom, Inc., San Diego, CA) at the Fudan-VARI Center for Genetic Epidemiology at Fudan University. Two duplicates and two water samples were included in each 96-well plate as PCR negative controls. Technicians who performed the genotyping assays were blinded to the identity of the samples. The overall genotyping rate was 98.8%. The average concordance rate between samples was 100% among the duplicated quality control samples.

### Statistical analysis

Quantitative variables were presented as mean ± standard deviation (SD). An independent *t* test was used to compare the differences between the means of continuous variables. Categorical variables were analyzed using the Chi-square test. The association of SNPs with urolithiasis was tested by a Cochran-Armitage trend test. We assumed a significant level of P = 6.25 × 10^−3^ (0.05/8). The odds ratios (OR) were calculated using non-susceptible allele as a reference. Multiple logistic regression analyses were applied to examine the relation of genotype and recurrent disease, diabetes mellitus and hypertension. Multiple linear regression analyses were used to test association between genotype and clinical parameters, including serum calcium, phosphorus, magnesium, creatinine, urea, uric acid, etc. with relevant covariates. We conducted association and QTL analyses using the PLINK-1.07 toolset. *P*-values were two-tailed. An α of 0.05 was used to claim statistical significance.

## Results

The clinical characteristics of case and control samples were shown in Table 1. There was no significant difference in the distribution of BMI, serum calcium, phosphorus, magnesium, creatinine, and uric acid among the patients and controls.

The genotype frequencies of eight SNPs among case and control subjects were distributed in accordance with the Hardy-Weinberg equilibrium. For the 8 polymorphic SNPs, none of them were significantly associated with urolithiasis risk in Chinese Han population at P < 6.25 × 10^−3^ threshold (Table 2). Next, we examined the association of these SNPs with several clinical parameters. The C allele of rs100597 was significantly associated with recurrent urolithiaisis disease (OR = 0.6652, P = 0.04864) and diabetes mellitus (OR = 0.5526, P = 0.04308) (Table 3). As shown in Table 4, the A allele of rs12669187 was significantly correlated with increased level of serum magnesium (P = 0.00929). The C allele of rs1000597 was associated with higher levels of serum creatinine (P = 0.04634), uric acid (P = 0.002559), calcium (P = 0.04641) and lower urine pH level (P = 0.0179). The T allele of rs4142110 was correlated with higher levels of serum magnesium (P = 0.02905), phosphorus (P = 0.03275), and lower serum AKP level (P = 0.04815). In addition, there was a significant association between the G alleles of rs4293393 and higher serum CO_2_ level (P = 0.02437).

## Discussion

Urabe *et al*.^6^ conducted a GWAS using a total of 5892 nephrolithiasis cases and 17809 controls of Japanese population and found three novel loci for nephrolithiasis, including rs11746643 on 5q35.3, rs100597 on 7p14.3 and rs4142110 on 13q14.1. Yasui *et al*.^7^ performed a replication study for these three loci and their results have reported that rs12654812 on 5q35.3, rs12669187 on 7p14.3 and rs7981733 on 13p14.1 were significantly associated with nephrolithiasis. In our study, however, we found that none of these eight reported SNPs were significantly associated with urolithiasis risk in Chinese Han population. Although Japan and Chinese Han populations are close each other, there showed large genetic difference between those two ethnics. Similar trends were also observed in association studies of *VDR* gene polymorphisms in patients with urolithiasis. *TaqI* (rs731236) SNP, located near the 3′ end of the *VDR* gene, has been shown to affect gene expression through regulation of mRNA stability. Aykan *et al*.^10^ reported that the risk allele for *TaqI* SNP was significantly associated with recurrent urolithiasis in a Caucasian population. However, in the study of Cakir *et al*.^11^, no significant difference was found between patients with nephrolithiasis and the control group for *TaqI* in Turkish population.

The *SLC34A1* gene located in the *RGS14*-*SLC34A1*-*PFN3*-*F12* region and it encodes NPT2a which is a member of the type IIa sodium-phosphate co-transporter family^12^. In kidney, the NPT2a protein resides in the apical membrane of proximal tubular cells, responsible for reclaiming most of the filtered phosphate load in a rate-limiting manner and it is essential in maintaining phosphate homeostasis. It was reported that mutations in *SLC34A1* gene may cause hypophosphatemia and urinary phosphate loss in patients with nephrolithiasis or osteoporosis^13^. In knockout mice, severe renal phosphate wasting, hypercalciuria and skeletal abnormalities were observed^14^. Previous GWAS reports have demonstrated the association of variations on *SLC34A1* locus with CKD^15^ and serum phosphorus concentration^16^. In a GWAS of nephrolithiasis in Japanese population, SNP rs11746443, located upstream of *SLC34A1* gene, was identified as one of the three novel susceptible nephrolithiasis loci. Moreover, the risk allele of SNP rs11746443 was found to be associated with the reduction of estimated glomerular filtration rate (eGFR) by QTL analyses. However, they did not find a significant association between rs11746443 and serum phosphorus^6^. In the replication study, rs12654812, located near *SLC34A1*, showed significant association with nephrolithiasis.

The previous GWAS study in Japanese population identified that SNP rs1000597, located between *FAM188B* gene and *AQP1* gene on chromosome 7p14.3, was significantly associated with nephrolithiasis^6^. In the replication study, SNP rs12669187, located on *FAM188B* gene, also showed a significant association with nephrolithiasis^7^. The role of *FAM118B* in the pathogenesis of nephrolithiasis has not been fully elucidated so far. *AQP1* gene codes for the water channels aquaporin-1, which functions as a water channel, and is abundantly expressed in kidney^17^. It was reported that *AQP1* knockout-mice did not produce calculi, but exhibited reduced osmotic permeability in the renal proximal tube membrane and became severely dehydrated after water deprivation^18^. *AQP1* gene SNPs could impair water reabsorption in renal proximal tubules and may stimulate urine concentration in distal tubules. Therefore, the SNPs rs12669187 and rs1000597 are likely to be associated with the regulation of *FAM188B* and *AQP1* expression, which could affect the urine concentration and increase the risk of urolithiasis development.

*DGKH* gene is located on chromosome 13q14.1 and encodes for diacylglycerol kinase eta (DGKH), a ubiquitously expressed kinase which is highly expressed in brain. DGKH belongs to the DGK family and it is potentially associated with variety of chronic diseases such as dipolar disorders, type 2 diabetes mellitus, epilepsy and certain forms of carcinoma^19^,^20^,^21^. However, its involvement in kidney function has not been elucidated. A study indicated that DGK might be involved in transplasmalemmal calcium ion influx of platelets^22^. In GWAS study by Urabe *et al*.^6^, SNP rs4142110 on *DGKH* was one of the three novel loci for nephrolithiasis. In replication study by Yashui *et al*.^7^, SNP rs7981733 on *DGKH* is significantly associated with nephrolithiasis. In a recent study of *DGKH* in Chinese population by Xu *et al*.^8^, SNP rs4142110 was associated with risk of calcium oxalate stone, whilst SNP rs17646069, located within intronic region, was not associated with increased risk of calcium oxalate stone.

*UMOD* gene encodes for uromodulin, also known as Tamm-Horsfall protein, which is an inhibitor of calcium-phosphate precipitation. The protein is a glycosylphosphatidylinositol-anchored glycoprotein mainly synthesized in thick ascending limb of Henle (TALH)^23^. Uromodulin is the most abundant protein in urine of healthy people, and it has been reported to prevent bacteria from adhering to epithelial cells of kidney and to inhibit calcium oxalate crystal aggregation^24^,^25^. *UMOD* knockout-mice have been shown to have decreased creatinine clearance and be more prone to urinary tract infections^26^,^27^. SNP rs4293393 is located 550 basepairs upstream of *UMOD* gene on 16p12.3 and has been proved to be associated with nephrolithiasis in a GWAS in Icelandic and Dutch populations. In contrast to CKD, a significant association between the *UMOD* variant and reduced risk of nephrolithiasis was observed in their results^9^.

Our results demonstrated that the risk allele of rs12669187 on *FAM188B* was associated with a higher level of serum magnesium. Since the serum level of magnesium is usually well maintained by the kidney^28^, the higher serum magnesium level can partly be explained as a result of the impaired urine concentration impacted by rs12669187 on 7p14.3. Magnesium has an important role as a urolithiasis inhibitor that it competes with calcium in binding oxalates in urine, and the ratio of magnesium/calcium in urine can be used as an estimate of stone risk^29^. Therefore, the urine magnesium deficiency result from abnormal urine concentration may be associated with the risk of urolithiasis.

In our study, the risk allele of rs1000597 on *AQP1* may also promote development of urolithiasis through affecting the level of serum calcium, uric acid, and urinary pH. The kidney has a key role in the control of calcium balance, and hypercalcemia takes a significant place in pathogenesis of the calcium urolithiasis. It was reported that stone formers had a relative risk of abnormal calcemia and calciuria 9, 18 times more than that of non-stone formers, respectively^30^. Hypercalcemia with hypercalciuria may cause the occurrence of calcium urolithiasis by increasing the saturation of calcium salts in urine and by binding inhibitors of stone formation^31^. The association between calcium urolithiasis and serum uric acid has long been a subject of investigation. Nearly one-third of patients with calcium urolithiasis have hyperuricosuria. *In vitro* studies reported that the increasing level of urine uric acid promotes crystallization of calcium oxalate^32^. Several clinical studies showed that after the urate-lowering therapy with allopurinol, the serum and urinary uric acid level decreased, and the risk of recurrent calcium stone reduced^33^. Moreover, it was reported that the formation of various types of urolithiasis was strongly influenced by urinary pH level. Changes in systemic acid-base homeostasis could alter urinary excretion of citrate, which was an important inhibitor of calcium urolithiasis^34^.

Additionally, the risk allele of rs1000597 was correlated with higher level of serum creatinine. *AQP1* greatly expressed in glomerular endothelial cells. Bedford *et al*.^35^ first reported that changes in *AQP1* expression were associated with a number of kidney disease, including chronic glomerulonephritis, interstitial nephritis, nephrosclerosis and so on. Nephrolithiasis have previously been suggested as a potential risk factor for CKD^36^. Paradoxically, CKD could be protective against forming nephrolithiasis because of the substantial reduction in urine calcium excretion^37^. Although the mechanisms of that association have not been fully elucidated, our results indirectly suggested that the presence of risk allele of rs1000597 was associated with urolithiasis. In the study of Meydan *et al*.^38^, they found an increased prevalence nephrolithiasis in patients with diabetes mellitus compared with controls. Lieske *et al*.^39^ reported that diabetes mellitus could be a risk factor in the development of uric acid stones of the urinary tract. Our results indicated that patients with C allele of rs1000597 have lower risk of recurrent urolithiasis and diabetes mellitus. These results suggested that the risk allele of rs1000597 could have a protective effect on urolithiasis.

Also, the risk allele of rs4142110 was correlated with lower serum AKP level and higher levels of serum magnesium and phosphorus. The kidney expresses AKP, which hydrolyses pyrophosphate to inorganic phosphate. Pyrophosphate is an inhibitor of calcification and crystallization present in human urine, and calcium urolithiasis formers appear to show reduced urinary pyrophosphate excretion^40^. Therefore, serum AKP level may have impact on the lithogenic process in the kidney. Serum phosphate was also proved as an important risk factor of calcium urolithiasis. The renal phosphate leak theory has been proposed to explain calcium urolithiasis in recurrent stone formers. That theory suggested that increased phosphate excretion from renal tubulary system could result in a decrease in serum phosphate and hypercalciuria^41^. Since rs4142110 was previously reported to be correlated with risk of hypercalciuria^8^, we speculated that the risk factor of rs4142110 could also contribute to renal phosphate leakage. However, the importance of renal phosphate leak theory in the pathogenesis of calcium stone formation is controversial. In the study of Dagnone *et al*.^42^, they concluded that serum phosphate did not appear to be an independent risk factor for the recurrence of urolithiasis, or a reliable early predictor of urolithiasis.

In the GWAS in Icelandic and Dutch populations, rs4293393 near *UMOD* was reported to influence the adaption of the kidney to age-related risk factors of kidney diseases, including hypertension and diabetes. The variation also showed associations with serum urea and uric acid in stone-formers^9^. In our study, the risk allele of rs4293393 was correlated with higher serum CO_2_. Since the serum level of CO_2_ generally falls as GFR decreases in CKD^43^, the risk allele of rs4293393 may not directly correlated with CKD. Moreover, since serum CO_2_ level was negatively correlated with the risk of uric acid stone^44^, our results indirectly suggested that the risk allele of rs4293393 was correlated with urolithiasis.

Although none of these eight reported SNPs were significantly associated with the risk of urolithiasis in Chinses Han population in the present study, we believed that negative results we obtained hereof were still of importance. Those results can possibly be explained by the racial disparities in lithogenic mechanisms between Chinese and Japanese ethnics. In east asia, although Japanese and Chinese Han ethnicity are very close each other, the genetic heterogeneity varies even to a greater extent than we could perceive via appearace, which warranted that even minor racial disparity needed for individual validation. Therefore, a GWAS in Chinese Han population is urgently needed, which will enable us to find more ethnic specific SNPs to study in the future. Nevertheless, most of the risk alleles of these SNPs were proved to be associated with abnormal serum electrolytic metabolism in patients with urolithiasis, which suggested that there could exist a gap between electrolytic metabolism and the final process of urinary stone formation. We believed that our results would contribute to a better understanding of the pathogenesis of urolithiasis, and further analysis is necessary to determine the role of these variations in the development of urolithiasis.

There are several limitations in the present study. First, the sample size of this study was relatively small, which may affect the statistical power to detect the possible risk for the SNPs of urolithiasis in Chinese Han population. We intended to address this limitation by expanding sample sizes in our future studies to further elucidate the impact of these loci on urolithiasis susceptibility. Second, the selection of SNPs was biased and other SNPs that may potentially be related to calcium urolithiasis were not included in our investigation. Most SNPs that we validated in the present study were selected from previous GWAS studies, which achieved the genome-wide significance threshold of P < 5 × 10^−8^. We plan to include more SNPs with a threshold for significance of P < 5 × 10^−7^ on those susceptible loci in our future studies. Morever, the limitation of lack of urine electrolytes data could not be easily ignored.

## Conclusion

We found none of these eight reported SNPs were significant associated with urolithiasis risk in Chinese Han population, which suggested that differences could exist in the mechanisms of calcium urolithiasis between Chinese and Japanese Ethnics. However, the risk alleles of these SNPs were proved to be associated with the electrolytes metabolism that may result in the formation of urolithiasis.

## Additional Information

**How to cite this article**: Wang, L. *et al*. Association Study of Reported Significant Loci at 5q35.3, 7p14.3, 13q14.1 and 16p12.3 with Urolithiasis in Chinese Han Ethnicity. *Sci. Rep.* **7**, 45766; doi: 10.1038/srep45766 (2017).

**Publisher's note:** Springer Nature remains neutral with regard to jurisdictional claims in published maps and institutional affiliations.

## Figures and Tables

**Table 1:** Baseline characteristic of the study population.

Parameter	Cases	Controls	*P* value
Age (years)	50.81 ± 13.65	49.98 ± 12.65	0.317
Gender (male/female)	441/183	705/303	0.753
BMI (kg/m^2^)	24.42 ± 3.35	24.28 ± 3.26	0.545
Stone frequency (primary/recurrence)	464/160	—	—
Serum calcium (mmol/L)	2.29 ± 0.13	2.27 ± 0.10	0.086
Serum phosphorus (mmol/L)	1.14 ± 0.20	1.14 ± 0.18	0.598
Serum magnesium (mmol/L)	0.89 ± 0.78	0.89 ± 0.75	0.389
Serum creatinine (μmol/L)	88.71 ± 43.24	83.64 ± 23.51	0.059
Serum uric acid (mmol/L)	0.361 ± 0.090	0.353 ± 0.077	0.158

**Table 2:** Results of association analysis for calcium urolithiasis in Chinese Han population.

Chr no.	SNP	Alleles		Cases				Controls				*P*	OR	95%CI
		Minor	Major	n(11)^a^	n(12)	n(22)	MAF	n(11)	n(12)	n(22)	MAF			
5	rs12654812	A	G	53	267	334	0.2852	82	373	553	0.2664	0.2644	1.091	(0.9362–1.271)
5	rs11746443	A	G	25	188	433	0.1842	39	271	698	0.1731	0.4499	1.071	(0.8966–1.279)
7	rs12669187	A	G	5	113	536	0.09404	11	171	825	0.09583	0.8421	0.9762	(0.7704–1.237)
7	rs1000597	C	T	7	174	473	0.1437	25	238	745	0.1429	0.9719	1.004	(0.8221–1.225)
13	rs7981733	T	C	92	296	266	0.367	147	482	379	0.3849	0.2688	0.9222	(0.799–1.065)
13	rs4142110	T	C	138	307	207	0.4471	211	513	284	0.4638	0.3466	0.9351	(0.8133–1.075)
13	rs17646069	C	T	25	174	455	0.1713	30	278	699	0.1678	0.7291	1.033	(0.861–1.239)
16	rs4293393	G	A	6	96	552	0.08257	5	141	860	0.07505	0.4393	1.107	(0.8562–1.43)

Abbreviatios: Chr, chromosome; CI, confident interval; MAF, minor allele frequency; OR, odds ratio; SNP, single-nucleotide polymorphism. ^a^n(11), number of subjects with homozygous genotypes for the minor allele; n(12), number of subjects with heterozygous genotypes; n(22), number of subjects with homozygous genotypes for the major allele.

**Table 3:** Multiple logistic regression analyses for clinical parameters.

SNP	Recurrent disease			Hypertension			Diabetes mellitus		
	OR	Stat^b^	*P*	OR	Stat	*P*	OR	Stat	*P*
rs12654812	1.269	1.691	0.09091	0.8011	−1.624	0.1043	0.6959	−1.793	0.07289
rs11746443	1.224	1.248	0.2121	0.8413	−1.09	0.2756	0.6278	−1.878	0.06038
rs12669187	0.7481	−1.224	0.221	1.07	0.3312	0.7405	0.6659	−1.229	0.2191
rs1000597	0.6652	−1.972	**0.04864**	1.074	0.3998	0.6893	0.5526	−2.023	**0.04308**
rs7981733	0.9086	−0.7118	0.4766	0.8309	−1.475	0.1403	0.8124	−1.16	0.2462
rs4142110	0.8524	−1.23	0.2188	0.8485	−1.369	0.1711	0.7588	−1.613	0.1067
rs17646069	0.8108	−1.186	0.2355	0.8847	−0.7671	0.443	1.065	0.2913	0.7708
rs4293393	1.187	0.7324	0.4639	1.439	1.684	0.09225	0.7577	−0.8038	0.4215

Abbreviations: SNP, single-nucleotide polymorphism; OR, odds ratio. ^b^Stat, Coefficient t-statistic.

**Table 4:** Multiple linear regression analyses for clinical parameters.

	rs12654812			rs11746443			rs12669187			rs1000597			rs7981733			rs4142110			rs17646069			rs4293393		
	Beta^c^	Stat	P	Beta	Stat	P	Beta	Stat	P	Beta	Stat	P	Beta	Stat	P	Beta	Stat	P	Beta	Stat	P	Beta	Stat	P
BMI	−0.3011	−1.428	0.1539	−0.0202	−0.08218	0.9345	0.1279	0.391	0.6959	−0.07357	−0.26	0.795	−0.215	−1.097	0.2731	−0.1895	−1.002	0.3169	0.174	0.7006	0.4838	−0.08073	−0.2276	0.82
GFR	−3.45	−1.433	0.1524	−3.673	−1.312	0.1902	0.4899	0.1312	0.8957	−0.7119	−0.2204	0.8257	1.38	0.6162	0.538	1.146	0.5312	0.5955	−1.294	−0.4559	0.6486	−0.4917	−0.1214	0.9034
Serum creatinine	1.031	0.3771	0.7063	−2.945	−0.9237	0.356	6.133	1.451	0.1472	7.288	1.996	**0.04634**	−2.922	−1.152	0.2498	−2.879	−1.175	0.2404	−2.389	−0.7428	0.4579	1.182	0.2574	0.7969
Serum urea	0.05685	0.4627	0.6437	−0.1035	−0.7205	0.4715	0.02246	0.118	0.9061	0.2336	1.422	0.1557	−0.07287	−0.6388	0.5232	−0.1946	−1.772	0.07694	0.05925	0.4103	0.6817	0.07602	0.3689	0.7123
Serum uric acid	0.00371	0.6547	0.5129	0.006474	0.9804	0.3273	0.009673	1.103	0.2703	0.02282	3.029	**0.002559**	0.003489	0.6632	0.5075	−5.751E-5	−0.01133	0.991	0.001978	0.2967	0.7668	0.00165	0.1734	0.8624
Serum sodium	0.2241	1.398	0.1626	0.2507	1.343	0.1797	0.406	1.636	0.1023	0.26	1.21	0.2268	−0.1137	−0.7628	0.4459	−0.1457	−1.013	0.3113	0.04915	0.2602	0.7948	0.3523	1.309	0.1911
Serum potassium	0.02514	1.044	0.2967	−0.001063	−0.03782	0.9698	0.002187	0.05863	0.9533	0.001507	0.04668	0.9628	−0.02443	−1.093	0.2748	−0.006205	−0.288	0.7734	0.02389	0.8446	0.3986	−0.01146	−0.2838	0.7767
Serum calcium	0.008207	1.019	0.3087	0.005057	0.5384	0.5905	0.0131	1.051	0.2938	0.02147	1.996	**0.04641**	−0.00415	−0.5543	0.5795	0.007013	0.9719	0.3315	−0.00852	−0.8936	0.3719	0.004885	0.3616	0.7178
Serum magnesium	0.004134	0.8325	0.4055	−0.002504	−0.431	0.6666	0.02019	2.61	**0.00929**	0.01309	1.953	0.05135	0.006842	1.479	0.1397	0.009679	2.188	**0.02905**	−0.007517	−1.259	0.2086	0.003918	0.4642	0.6427
Serum phosphorus	0.0003316	0.02672	0.9787	−0.006062	−0.4191	0.6753	−0.0001675	−0.00872	0.993	−0.01015	−0.6111	0.5414	0.01886	1.64	0.1014	0.0237	2.14	**0.03275**	−0.01304	−0.8935	0.3719	0.0192	0.9219	0.3569
Serum AKP	0.9711	0.5909	0.5548	0.5444	0.284	0.7765	0.7954	0.3125	0.7548	0.225	0.1022	0.9186	−1.676	−1.099	0.2722	−2.903	−1.98	**0.04815**	0.612	0.3164	0.7518	0.3631	0.1315	0.8954
Serum γGT	0.384	0.2261	0.8212	−0.3414	−0.1723	0.8633	−2.793	−1.064	0.288	−0.2218	−0.09751	0.9224	0.6422	0.4073	0.6839	1.77	1.164	0.2448	1.048	0.5247	0.6	−0.7022	−0.2463	0.8056
Serum albumin	−0.1777	−0.6644	0.5067	0.1171	0.3757	0.7073	−0.09469	−0.2288	0.8191	0.01961	0.05476	0.9564	−0.001527	−0.006131	0.9951	−0.03346	−0.1399	0.8888	−0.03822	−0.1208	0.9039	0.4697	1.049	0.2946
Serum total protein	0.2245	0.571	0.5682	0.2987	0.6543	0.5132	0.6176	1.016	0.3099	0.2906	0.5523	0.5809	−0.1273	−0.3479	0.728	0.176	0.5006	0.6168	−0.47	−1.011	0.3125	0.9857	1.499	0.1345
Serum carbon dioxide	−0.03645	−0.2233	0.8234	−0.02726	−0.1447	0.885	0.1741	0.689	0.4911	0.02588	0.1183	0.9058	0.259	1.712	0.08737	0.238	1.629	0.1039	0.2492	1.299	0.1944	0.616	2.257	**0.02437**
Urine SG	0.0003518	0.4984	0.6184	−0.0002127	−0.2584	0.7962	−0.001372	−1.256	0.2095	−0.001043	−1.104	0.2701	6.706E-5	0.1023	0.9186	−0.0001405	−0.222	0.8244	5.552E-5	0.06683	0.9467	−0.0009654	−0.8148	0.4155
Urine PH	0.00872	0.2453	0.8063	0.03557	0.8621	0.389	−0.09692	−1.766	0.07795	−0.1125	−2.374	**0.0179**	−0.01377	−0.4171	0.6768	−0.02728	−0.8562	0.3922	−0.04108	−0.9828	0.3261	0.09505	1.596	0.1111

Abbreviations: BMI, body mass index; GFR, glomerular filtration rate; AKP, alkaline phosphatase; γGT, γ-glutamyl transferase; SG, specific gravity. ^c^Beta, Regression coefficient.
